# *Edwardsiella tarda*-Induced Inhibition of Apoptosis: A Strategy for Intracellular Survival

**DOI:** 10.3389/fcimb.2016.00076

**Published:** 2016-07-14

**Authors:** Ze-jun Zhou, Li Sun

**Affiliations:** ^1^Key Laboratory of Experimental Marine Biology, Institute of Oceanology, Chinese Academy of SciencesQingdao, China; ^2^Laboratory for Marine Biology and Biotechnology, Qingdao National Laboratory for Marine Science and TechnologyQingdao, China; ^3^University of Chinese Academy of SciencesBeijing, China

**Keywords:** *Edwardsiella tarda*, invasion, intracellular replication, apoptosis, zebrafish

## Abstract

*Edwardsiella tarda* is a Gram-negative bacterial pathogen that can infect a wide range of freshwater and marine fish. One salient feature of *E. tarda* is the ability to survive and replicate in various host cells. In this study, we observed that *E. tarda* replicated robustly in the zebrafish cell line ZF4, and that *E. tarda*-infected cells exhibited no detectable signs of apoptosis. Global transcriptome analysis and quantitative real-time RT-PCR revealed that *E. tarda* infection generally significantly downregulated pro-apoptotic genes and upregulated anti-apoptotic genes. To investigate the role of apoptosis in *E. tarda* infection, two upregulated anti-apoptotic genes (*Fech* and *Prx3*) and two downregulated pro-apoptotic genes (*Brms1a* and *Ivns1a*) were overexpressed in zebrafish. Subsequent infection study showed that *Fech* and *Prx3* overexpression significantly promoted *E. tarda* dissemination in and colonization of fish tissues, while *Brms1a* and *Ivns1a* overexpression significantly reduced *E. tarda* dissemination and colonization. Consistently, when *Fech* and *Prx3* were knocked down in zebrafish, *E. tarda* infection was significantly inhibited, whereas *Brms1a* and *Ivns1a* knockdown significantly enhanced *E. tarda* infection. These results indicate for the first time that *E. tarda* prevents apoptosis in teleost as a strategy for intracellular survival, and that some putative apoptotic genes of teleost function in the apoptosis pathway probably in a manner similar to that in mammalian systems.

## Introduction

Apoptosis is a highly programmed cell death process that occurs regularly in multicellular organisms (Elmore, 2007). It differs from other forms of programmed cell death, such as pyroptosis, necroptosis, autophagy, and NETosis, and is characterized by DNA fragmentation, nuclear condensation, cytoplasmic shrinkage, caspase activation, and cell death without lysis or damage to adjacent cells (Elmore, 2007; Linkermann et al., 2014; Tait et al., 2014). Apoptosis is activated by intrinsic and extrinsic pathways, and is typically observed in response to infection, which leads to removal of an intracellular niche for microbes, release of molecules with direct microbicidal activity, and propagation of an inflammatory response (Stephenson et al., 2016). Thus, it is not surprising that prevention of apoptosis provides a survival advantage for microbial pathogens as it enables the microbes to replicate intracellularly and evade the inflammation and antimicrobial effects outside the host cells (Faherty and Maurelli, 2008; Raymond et al., 2013). Recent studies indicate that bacterial pathogens have evolved several ways to inhibit apoptosis, such as by protecting the mitochondria and preventing cytochrome *c* release, by activating cell survival pathways, and by preventing caspase activation (Rudel et al., 2010; Siamer and Dehio, 2015).

*Edwardsiella tarda* is a Gram-negative bacterial pathogen of the Enterobacteriaceae family. It has a broad host range and can inhabit in humans, animal, and fish (Leung et al., 2012). In aquaculture, *E. tarda* is recognized as a severe pathogen and can cause a systemic disease, edwardsiellosis, to many freshwater and marine fish (Park et al., 2012). In addition to fish, *E. tarda* is also a human pathogen and known to cause bacteremia in humans (Hirai et al., 2015). One distinct virulence feature of *E. tarda* is a strong ability to stay alive and replicate in host phagocytes during infection (Rao et al., 2001; Ishibe et al., 2008; Cheng et al., 2010). Intracellular survival of *E. tarda* has also been observed in mammalian cell lines and fish cell lines derived from flounder and fathead minnow (Okuda et al., 2006, 2008; Wang et al., 2013). It has been reported that *E. tarda* was able to escape from the endocytic vacuole and replicate within the cytoplasm, and that *E. tarda* could spread by lysing the plasma membrane after several rounds of replication (Strauss et al., 1997). In addition, many virulence-associated factors/systems, such as type VI secretion system and hemolysin, are required for *E. tarda* to enter host cells (Strauss et al., 1997; Leung et al., 2012). However, the mechanism through which *E. tarda* manipulates host cell signaling pathway remains unknown.

In the current study, we aimed to examine the pathogenic mechanism of *E. tarda* associated with intracellular survival. For this purpose, we first conducted a transcriptome analysis to investigate the global gene expression profile of *E. tarda* following infection of a zebrafish cell line. The results of transcriptome analysis suggested to us the possibility of *E. tarda*-induced inhibition of apoptosis, which was subsequently confirmed by a series of experiments.

## Materials and methods

### Ethics

Experiments involving live animals were conducted in accordance with the “Regulations for the Administration of Affairs Concerning Experimental Animals” promulgated by the State Science and Technology Commission of Shandong Province. The study was approved by the ethics committee of Institute of Oceanology, Chinese Academy of Sciences.

### Fish

Clinically healthy zebrafish were purchased from a commercial fish farm and maintained at 24°C in a zebrafish cultivation system. Before experiment, fish were verified to be free of bacteria in kidney and spleen by plate count as reported previously (Li and Zhang, 2015). Fish were euthanized with an overdose of tricaine methanesulfonate (Sigma, St. Louis, USA) before tissue collection.

### Intracellular bacterial replication

*E. tarda* TX01 (Zhang et al., 2008) was cultured in Luria-Bertani broth (LB) medium to an OD_600_ of 0.8. The cells were washed with PBS and resuspended in PBS to 1 × 10^8^ CFU/ml. ZF4 cells (American type culture collection, USA), a zebrafish cell line, were cultured at 24°C in 96-well cell culture plates (~10^5^ cells/well) with DMEM/F-12 medium (GIBCO, Invitrogen, Carlsbad, USA) containing penicillin (60 μg/ml), streptomycin (100 μg/ml) and 10% fetal bovine serum (FBS). *E. tarda* suspension was added to FG cells to a MOI of 10:1. The plate was incubated at 24°C for 3 h and washed five times with PBS. The cells were treated with gentamicin (200 μg/ml) for 2 h to kill extracellular bacteria. After treatment, the cells were washed three times with PBS and cultured in fresh DMEM/F-12 medium for 3, 6, 12, 18, and 24 h. At each time point, the cells were lysed, and viable bacteria were detected by plate count as above. The assay was performed three times, each time with three replicates.

### Preparation of rat antibody against *E. tarda*

Rat anti-*E. tarda* antibody was prepared as reported previously (Yu et al., 2013; Hu et al., 2014). Briefly, *E. tarda* TX01 was cultured in LB medium to an OD_600_ of 0.8 and harvested by centrifugation at 4°C. The bacterial cells were washed with PBS for three times and resuspended in PBS. Three adult rats (purchased from the Institute for Drug Control, Qingdao, China) were immunized via subcutaneous injection with 1 × 10^5^ CFU *E. tarda*. The rats were boosted at 20 and 30 days after the initial immunization. The rats were bled at 12 days after the last boost, and sera were obtained from the blood. The specificity and titer of serum antibody were determined by Western immunoblot and enzyme-linked immunosorbent assay as reported previously (Hu et al., 2014). The antibody was aliquoted and stored at −80°C.

### Cellular apoptosis

ZF4 cells were infected with *E. tarda* as above or treated with cisplatin (Beyotime, Shanghai, China) at the final concentration of 100 μM for 12 or 24 h. Cisplatin is a widely used anticancer drug that can cause the DNA damage, induce cytochrome *c* release and subsequent caspase activation and apoptosis (Mandic et al., 2003). The control cells were treated with PBS. The cells were then used for (i) cellular DNA extraction. The DNA was extracted with DNA Extraction Kit (Beyotime, Shanghai, China) and subjected to electrophoresis analysis in a 1.0% agarose gel; (ii) microscopic observation. The cells were washed with PBS and incubated with 4% paraformaldehyde for 30 min. After incubation, rat antibody against *E. tarda* (1/1000 dilution), which had been prepared previously (as described above) and stored in the laboratory, was added to the cells. The cells were incubated at 28°C for 2 h and washed 3 × with PBS. Fluorescein isothiocyanate (FITC)-labeled goat anti-rat IgG (Bioss, Beijing, China; 1/1000 dilution) was added to the cells. The cells were incubated at 37°C for 1 h. The cells were washed twice with PBS and stained for 5 min at room temperature in Hoechst 33258 (1 μg/ml in PBS; Beyotime, Shanghai, China). After washing, the cells were observed with fluorescent microscope (Nikon E800, Japan); (iii) TUNEL assay. The cells were incubated with 4% paraformaldehyde and then treated with *E. tarda* antibody as above. The cells were incubated at 28°C for 2 h and washed 3 × with PBS. Cy3-labeled goat anti-rat IgG (Bioss, Beijing, China; 1/1000 dilution) was added to the cells. After incubation at 37°C for 1 h, the cells were examined using an *in situ* Cell Death Detection Kit (Roche Diagnostics, Mannheim, Germany) according to the manufacturer's instruction; (iv) Annexin V-FITC/PI assay. The washed cells were treated with FITC-conjugated annexin V and propidium iodide (PI) by using annexin V-FITC and PI Cell Apoptosis Detection Kit (Majorbio Biotech, Shanghai, China) according to the manufacturer's instruction. The cells were then subjected to flow cytometry using a FACSort Flow Cytometer (BD Biosciences, San Jose, California, USA) equipped with FlowJo software (Tree Star Inc., San Carlos, California, USA) for data analysis; (v) analysis of caspase activity. The activities of caspase 3, 8, and 9 were detected using Caspase 3/8/9 Activity Assay Kits (Beyotime, Shanghai, China) according to the manufacturer's instruction. All experiments were performed three times, each time with three replicates.

### Transcriptome analysis

Transcriptome analysis was performed as reported previously (Yang et al., 2012). Briefly, ZF4 cells were infected with *E. tarda* as above, and at 0 and 12 h post-infection (hpi), total samples were subsequently stored at −80°C until RNA extraction for RNA-seq analysis. The RNA samples described above were subjected to cDNA library construction and deep sequencing and analysis performed by LC Sciences LLC, USA. The genome sequence data of zebrafish in NCBI databank (*Danio rerio* reference genome: GRCz10) were used for transcriptome study. Raw data of RNA-seq were deposited in NCBI database under submission number GSE81773.

### Quantitative real-time reverse transcription-PCR (qRT-PCR)

ZF4 cells were infected with *E. tarda* as above, and at 0, 6, 12, 24, and 48 h post-infection (hpi), total RNA was extracted from the cells with EZNA Total RNA Kit (Omega Bio-tek, Doraville, USA). The RNA was treated with RNase-free DNaseI (TaKaRa, Dalian, China). One microgram of RNA was used for cDNA synthesis with the Superscript II reverse transcriptase (Invitrogen, Carlsbad, USA). qRT-PCR was carried out in an Eppendorf Mastercycler (Eppendorf, Hamburg, Germany) using SYBR ExScript qRT-PCR Kit (Takara, Dalian, China) as described previously (Zhang et al., 2013). The genes examined and the primers used for qRT-PCR are listed in Table S1. The expression level of the genes was analyzed using comparative threshold cycle method (2^−ΔΔCT^) with beta actin (ACTB) as an internal control as reported previously (Yang et al., 2012). The experiment was performed three times, each time with three replicates.

### Construction of pFech, pPrx3, pBrms1a, and pIvns1a

The eukaryotic expression plasmids pFech, pPrx3, pBrms1a, and pIvns1a, which express His-tagged Fech, Prx3, Brms1a, and Ivns1a respectively, were constructed as follows. The coding sequences of Fech, Prx3, Brms1a, and Ivns1a were amplified by PCR with the primer pairs Fech-OE-F/Fech-OE-R, Prx3-OE-F/Prx3-OE-R, Brms1a-OE-F/Brms1a-OE-R, and Ivns1a-OE-F/Ivns1a-OE-R, respectively (Table S1), which were designed based on the sequences of zebrafish Fech, Prx3, Brms1a, and Ivns1a, respectively (GenBank accession numbers: BC092711.1, BC092846.1, NM_001030102.2, and BC046068.1, respectively). The PCR products were ligated with the T-A cloning vector pEASY-Simple-T (TransGen Biotech, Beijing, China), and the recombinant plasmids were digested with EcoRV to retrieve the *Fech, Prx3, Brms1a*, and *Ivns1a* fragments. The fragments were inserted into pCN3 (Jiao et al., 2009) at the EcoRV site.

### Gene overexpression in zebrafish

Gene overexpression in fish was performed as reported previously (Wang and Sun, 2015). Briefly, endotoxin-free plasmid DNA was prepared using Endo-Free plasmid Kit (Omega Bio-Tek, Doraville, USA). The quality of the DNA was examined by determining *A*_260∕280_ and *A*_260∕230_ absorbance ratio using NanoDrop 2000 (Thermo scientific, USA) and by gel electrophoresis. The DNA was diluted in PBS to 600 μg/ml. Zebrafish (average 0.3 g) were divided randomly into five groups (24 fish/group) and injected i.m. with 5 μl pFech, pPrx3, pBrms1a, pIvns1a, pCN3, or PBS. Kidney and spleen were taken from the fish at 3 days post-plasmid administration. For PCR analysis of plasmid presence, DNA was extracted from the tissues with the TIANamp DNA Kit (Tiangen, Beijing, China). PCR detection of pFech, pPrx3, pBrms1a, pIvns1a, and pCN3 was performed with the primer pairs CNF1/Fech-OE-R, CNF1/Prx3-OE-R, CNF1/Brms1a-OE-R, CNF1/Ivns1a-OE-R, and CNF1/CNR1 respectively (Table S1). To examine transcription of plasmid-encoded *Fech, Prx3, Brms1a*, and *Ivns1a*, total RNA was extracted from the tissues as described above and used for RT-PCR with the primer pairs Fech-OE-F/His-R, Prx3-OE-F/His-R, Brms1a-OE-F/His-R, and Ivns1a-OE-F/His-R, respectively (Table S1). In each primer pair, the forward primer is specific to the target gene (i.e., *Fech, Prx3, Brms1a*, and *Ivns1a*), while the reverse primer is specific to the His-tag of the common backbone plasmid. As an internal control, RT-PCR was also performed the primers specific to β-actin. At 3 days post-plasmid administration, the fish were infected via i.m. injection with *E. tarda* (5 × 10^4^ CFU/fish). At 12, 24, and 48 h post-infection, kidney and spleen were taken under aseptic conditions and examined for bacterial numbers by plate count as reported previously (Li et al., 2015). The experiment was performed three times, each time with three replicates.

### Gene knockdown in zebrafish

Gene knockdown in fish was performed as reported previously (Wang and Sun, 2015). To select small interfering RNA (siRNA) for *Fech, Prx3, Brms1a*, and *Ivns1a* knockdown, three different siRNAs targeting each of these genes were inserted into the siRNA expression vector pRNAT-CMV3.1 (GenScript, Piscataway, USA) at BamHI/AlfII sites, resulting in four sets of plasmids, i.e., psiFech-1, psiFech-2, and psiFech-3 targeting *Fech*; psiPrx3-1, psiPrx3-2, and psiPrx3-3 targeting *Prx3*; psiBrms1a-1, psipsiBrms1a-2, and psiBrms1a-3 targeting *Brms1a*; psiIvns1a-1, psiIvns1a-2, and psiIvns1a-3 targeting *Ivns1a*. In addition, the plasmids psiCf, psiCp, psiCb, and psiCi, which express non-specific siRNAs for *Fech, Prx3, Brms1a*, and *Ivns1a* respectively, were constructed in the same fashion. The plasmids were purified endotoxin-free as described above. To examine the interfering efficiency of these siRNA plasmids, zebrafish were injected i.m. with each of the plasmids (3 μg/fish) or with PBS. At 3 and 5 days post-plasmid administration, spleen and kidney were taken under aseptic conditions and examined for the transcription of *Fech, Prx3, Brms1a*, and *Ivns1a* by qRT-PCR as described above. The plasmids with the strongest inhibitory effect on the target genes were re-named psiFech, psiPrx3, psiBrms1a, and psiIvns1a, respectively. This screening experiment was performed three times. The siRNA sequences expressed by psiFech, psiPrx3, psiBrms1a, and psiIvns1a are 5′-CAGGCATTCT GATGCTGAA-3′, 5′-GCTTGCTTC TCAATCAGCG-3′, 5′-GATCGGCATA GTATAGATA-3′, and 5′-GACCACAAA CAATACTTAC-3′ respectively. To examine the effect of *Fech, Prx3, Brms1a*, and *Ivns1a* knockdown on *E. tarda* infection, zebrafish were administered with psiFech, psiPrx3, psiBrms1a, psiIvns1a, psiCf, psiCp, psiCb, psiCi, or PBS (control) as above, and at 3 days post-plasmid administration, the fish were infected with *E. tarda* as above. At 12, 24, and 48 h post-infection, bacterial numbers in kidney and spleen were determined as above. The experiment was performed three times, each time with three replicates.

### Caspase 3 activity

Zebrafish were administered with various plasmids as above. At 3 days post-plasmid administration, spleen was taken under aseptic conditions and homogenized in lysis buffer according to the manufacture's protocol, and the activity of caspase 3 in spleen was then determined by Caspase 3 Activity Assay Kit (Beyotime, Shanghai, China).

### Statistical analysis

All statistical analyses were performed with analysis of variance (ANOVA) of the SPSS 15.0 package (SPSS Inc., Chicago, USA). In all cases, the significance level was defined as *P* < 0.05.

## Results

### *E. tarda* infection of ZF4 cells

**Figure 1:**
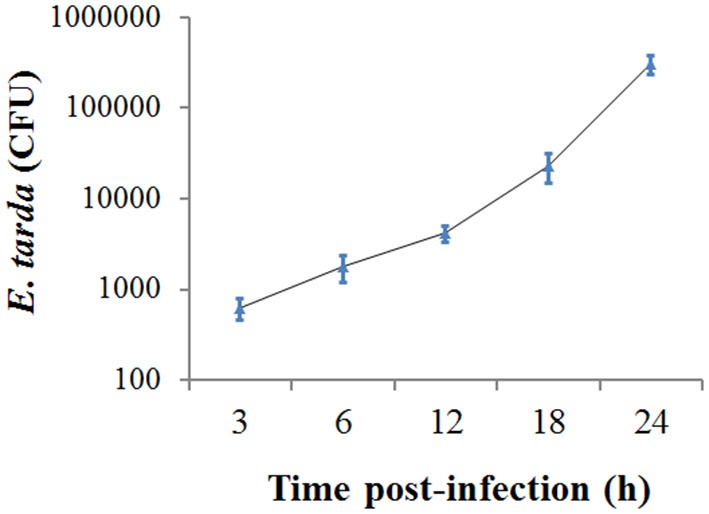
**Replication of ***Edwardsiella tarda*** inside ZF4 cells**. ZF4 cells were infected with *E. tarda* for 3 h, and extracellular bacteria were killed. The cells were then incubated further for various hours, and the number of intracellular bacteria was determined by plate count. Data are the means of three independent experiments and presented as means ± SEM.

When ZF4 cells were incubated with *E. tarda* for 3 h, bacterial invasion into the cells was detected. To examine the replicability of *E. tarda* inside ZF4, the extracellular bacteria were killed, and the cells were further incubated for 3, 6, 12, 18, and 24 h. The intracellular bacterial load was determined at each time point, which showed that the bacterial number increased almost linearly with time (Figure 1).

### Transcriptome analysis of *E. tarda*-induced gene expression profile in ZF4 cells

To investigate the effect of *E. tarda* infection on host gene expression, transcriptome analysis was conducted to examine the gene expression profiles of ZF4 before and after *E. tarda* infection. The criteria of a two-fold or greater change in expression and *p* < 0.01 were used to determine significantly upregulated/downregulated genes. The results showed that 948 genes exhibited significant changes in expression, including 576 upregulated genes and 372 downregulated genes (Figure S1; Additional file 1). KEGG pathway analysis of the 948 genes indicated that the top 10 enriched pathways were involved in lipid metabolism, apoptosis, MAPK signaling, spliceosome, carbohydrate metabolism, endocytosis, focal adhesion, PPAR signaling, Jak-STAT signaling, and cell cycle (Figure S2).

### Apoptosis status of *E. tarda*-infected cells

**Figure 2:**
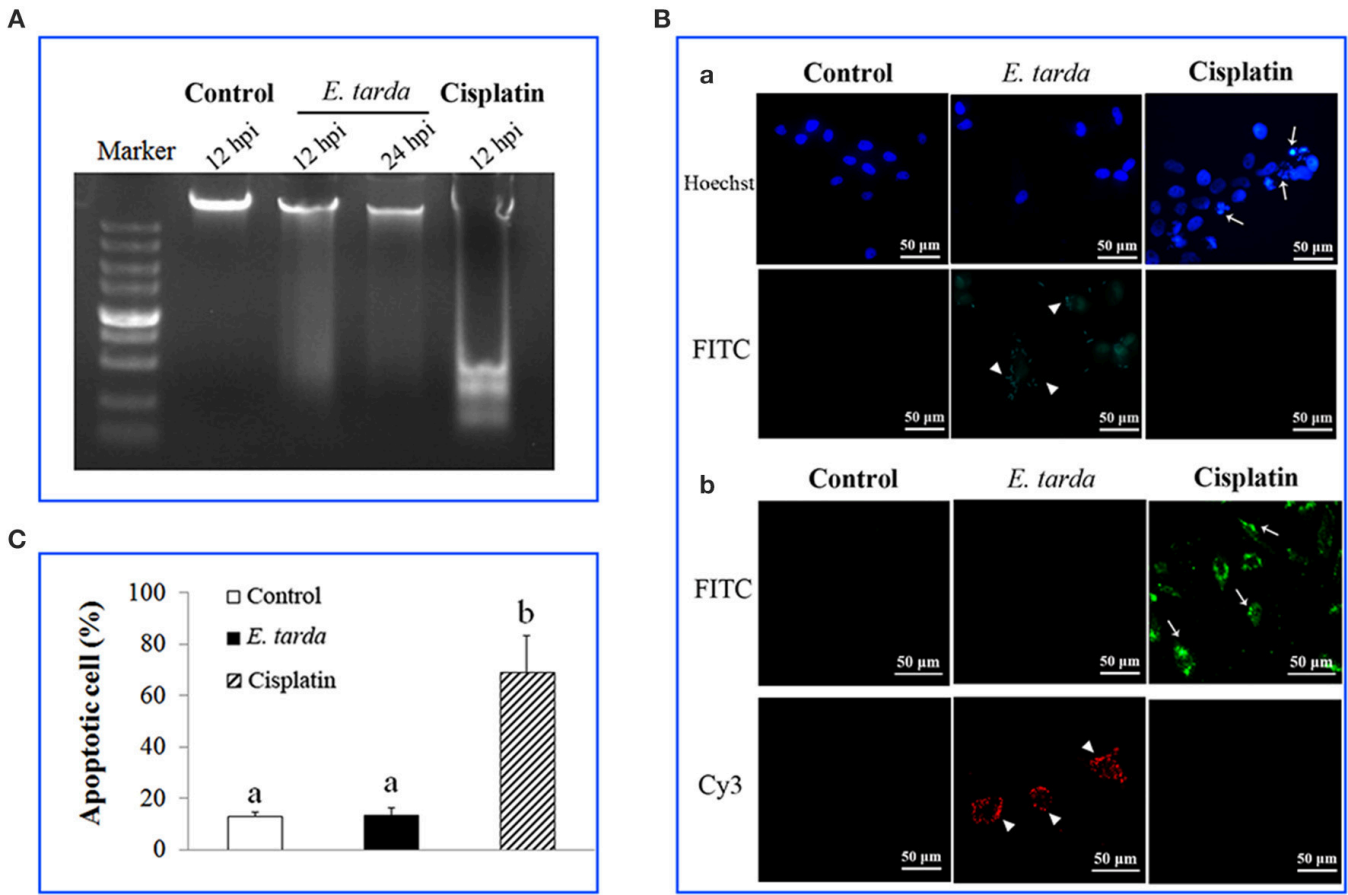
**Effect of ***Edwardsiella tarda*** infection on the apoptosis status of ZF4 cells. (A)** ZF4 cells were treated with *E. tarda* or cisplatin for different hours, and cellular DNA was subjected to agarose gel electrophoresis. **(B)** ZF4 cells were treated with *E. tarda* or cisplatin for 12 h, and apoptotic bodies **(Ba)** and TUNEL positive cells **(Bb)** were observed with a fluorescence microscope. *E. tarda* were detected by FITC-labeled antibody **(Ba)** or Cy3-labeled antibody **(Bb)**. Arrow heads indicate *E. tarda* cells; arrows indicate ZF4 cells. **(C)** ZF4 cells were treated with *E. tarda* or cisplatin as in **(B)**, and apoptosis of cells was assayed using Annexin V-FITC/PI assay. The experiment was performed three times, and significant differences in values are indicated by different letters.

Since, as shown above, the genes associated with apoptosis were markedly affected in expression by *E. tarda*, we examined the impact of *E. tarda* infection on the apoptosis of ZF4 cells. For this purpose, the cells were treated with *E. tarda* or cisplatin, which is known to trigger apoptosis, for 12 and 24 h. Subsequent analysis showed that DNA ladder was observed in cisplatin-treated cells but not in *E. tarda*-treated cells (Figure 2A). Likewise, fluorescence microscopy revealed that apoptotic bodies and TUNEL-positive staining were observed in cisplatin-treated cells but not in *E. tarda*-treated cells (Figure 2B). Annexin V-FITC/PI assay showed that the portion of apoptotic cells in cisplatin-treated ZF4 was significantly higher than that in the control cells, whereas the portion of apoptotic cells in *E. tarda*-treated ZF4 was similar to that of the control cells (Figure 2C). Consistently, caspase activity analysis indicated that the activities of caspase 3, 8, and 9 in *E. tarda*-infected ZF4 cells were comparable to those in the control cells (Figure S3).

### Expression of apoptosis-associated genes in *E. tarda*-infected cells

**Figure 3:**
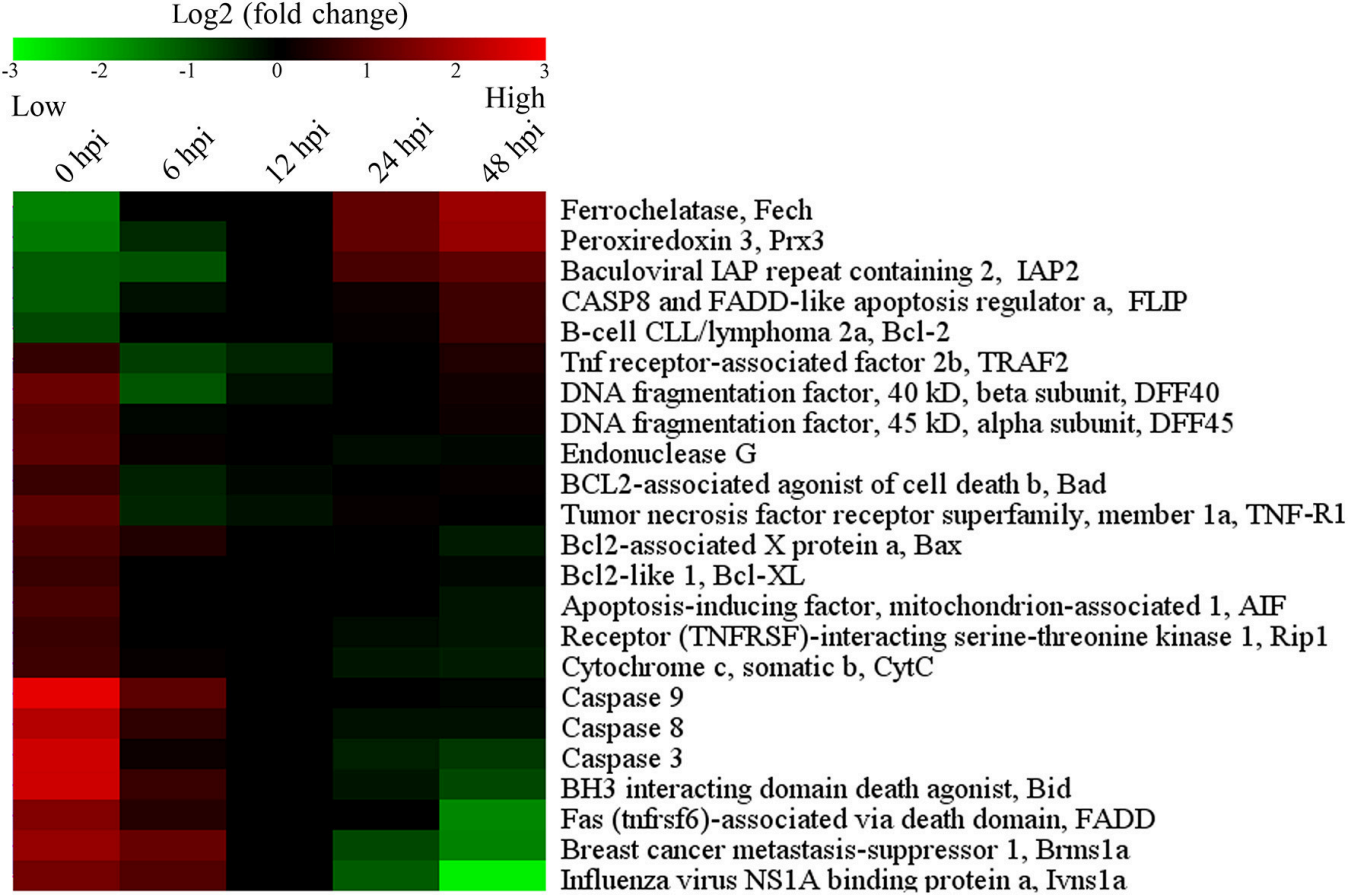
**Temporal expression profiles of apoptosis-associated genes shown in colored mosaic matrix**. ZF4 cells were infected with *E. tarda*, and the expression levels of apoptosis-associated genes were determined by quantitative real time RT-PCR at different hour post-infection (hpi). The expression levels were represented by different color bars.

To further examine the effect of *E. tarda* on apoptosis, qRT-PCR was performed to determine the mRNA levels of 23 apoptosis-associated genes in *E. tarda*-infected ZF4 at 0, 6, 12, 24, and 48 h post-infection (hpi). The results showed that the expression levels of the five anti-apoptotic genes (*Fech, Prx 3, IAP2, FLIP*, and *Bcl-2*) generally increased, with the highest induction occurring at 48 hpi (Figure 3; Table S2). In contrast, the 18 pro-apoptotic genes were downregulated during *E. tarda* infection, with the lowest expressions being observed with *DFF40, DFF45, TRAF2, Bad*, and *TNF-R1* at 6 hpi. In addition, the expression of caspase *3/8/9, FADD, Bid, CytC, Rip1, AIF, Bcl-XL, EndG, Bax, Brms1a*, and *Ivns1a* were decreased, with the lowest level of expression occurring at 48 hpi (Figure 3; Table S2).

### Overexpression of apoptosis-associated genes in zebrafish and its effect on *E. tarda* infection

#### Overexpression of Fech, Prx3, Brms1a, and Ivns1a in zebrafish

To examine the role of apoptosis on *E. tarda* infection, we selected, based on the above results, four apoptosis-associated genes regulated by *E. tarda*, i.e., *Fech, Prx3, Brms1a*, and *Ivns1a*, the first two being anti-apoptosis genes upregulated by *E. tarda*, while the last two being pro-apoptosis genes downregulated by *E. tarda*. Sequence alignment showed that zebrafish Fech, Prx3, Brms1a, and Ivns1a share 70.7, 70.2, 86.2, and 44.7%, respectively, overall sequence identities with their human counterparts. To create a condition of *Fech, Prx3, Brms1a*, and *Ivns1a* overexpression in zebrafish, the plasmids pFech, pPrx3, pBrms1a, and pIvns1a were constructed, which express His-tagged *Fech, Prx3, Brms1a*, and *Ivns1a*, respectively. Zebrafish were administered separately with each of the plasmids or with the control vector pCN3. At 3 days post-plasmid administration, PCR analysis showed that pFech, pPrx3, pBrms1a, pIvns1a, and pCN3 were present in the spleen and kidney of the fish administered with the respective plasmids but not in the fish administered with PBS (Figure S4A and data not shown), while RT-PCR showed that mRNA specific to plasmid-encoded *Fech, Prx3, Brms1a*, and *Ivns1a* were present in the spleen and kidney of the fish administered with pFech, pPrx3, pBrms1a, and pIvns1a respectively, but not in the fish administered with pCN3 or PBS (Figure S4B and data not shown). These results indicate that following administration into the host, pFech, pPrx3, pBrms1a, and pIvns1a were transported from the injection site into internal tissues, where the exogenous genes carried on the plasmids were expressed.

#### Effects of Fech, Prx3, Brms1a, and Ivns1a overexpression on *E. tarda* infection

**Figure 4:**
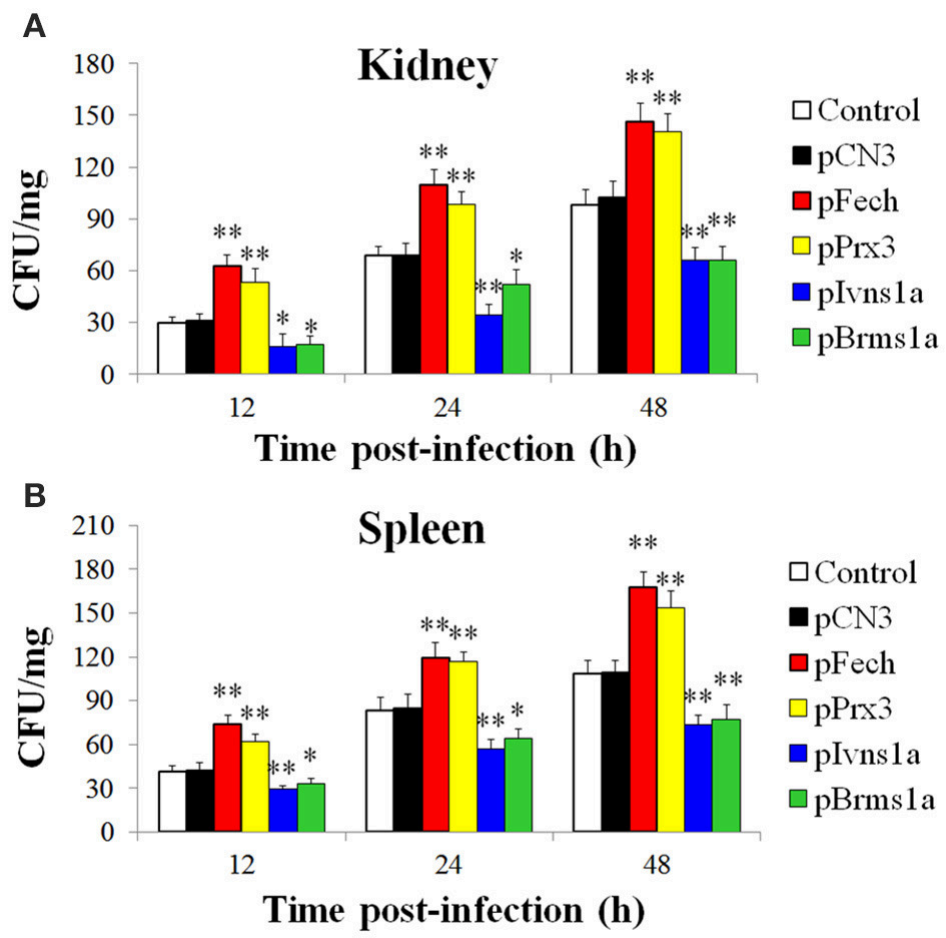
**Effect of overexpressing ***Fech***, ***Prx3***, ***Brms1a***, and ***Ivns1a*** on ***Edwardsiella tarda*** infection**. Zebrafish were administered with pFech, pPrx3, pBrms1a, pIvns1a, pCN3, or PBS (control) and challenged with *Edwardsiella tarda* at 3 days post-plasmid administration. Bacterial numbers in kidney **(A)** and spleen **(B)** were determined at various time points after infection. Data are the means of three independent experiments and presented as means ± SEM. ^*^P < 0.05, ^**^P < 0.01.

The fish administered with pFech, pPrx3, pBrms1a, pIvns1a, and pCN3 were challenged with *E. tarda*, and bacterial invasion into kidney and spleen was determined at 12, 24, and 48 hpi. The results showed that in both tissues and at all examined time points, the bacterial numbers in fish administered with pFech and pPrx3 were significantly higher than those in the control fish, whereas the bacterial numbers in fish administered with pBrms1a and pIvns1a were significantly lower than those in the control fish (Figure 4). In contrast, the bacterial burdens in pCN3-administered fish were comparable to those in the control fish.

### Knockdown of apoptosis-associated genes in zebrafish and its effect on *E. tarda* infection

#### Knockdown of Fech, Prx3, Brms1a, and Ivns1a

To further investigate the importance of *Fech, Prx3, Brms1a*, and *Ivns1a* to *E. tarda* infection, conditions of knockdown of these genes were created in zebrafish by administration into the fish the plasmids psiFech, psiPrx3, psiBrms1a, and psiIvns1a, which express specific siRNAs targeting *Fech, Prx3, Brms1a*, and *Ivns1a*, respectively. As controls, fish were also administered with psiCf, psiCp, psiCb, and psiCi, which express non-specific control siRNAs for *Fech, Prx3, Brms1a*, and *Ivns1a*, respectively. qRT-PCR revealed that at 3 and 5 d post-plasmid administration, the expression levels of *Fech, Prx3, Brms1a*, and *Ivns1a* in the kidney and spleen of the fish administered with psiFech, psiPrx3, psiBrms1a, and psiIvns1a were significantly lower than those in the control fish or in fish administered with psiCf, psiCp, psiCb, and psiCi (Figure S5). These results indicate that the siRNAs expressed by psiFech, psiPrx3, psiBrms1a, and psiIvns1a effectively interfered with the expression of *Fech, Prx3, Brms1a*, and *Ivns1a*, respectively, from the host genome.

#### Effects of fech, Prx3, Brms1a, and Ivns1a knockdowns on *E. tarda* infection

**Figure 5:**
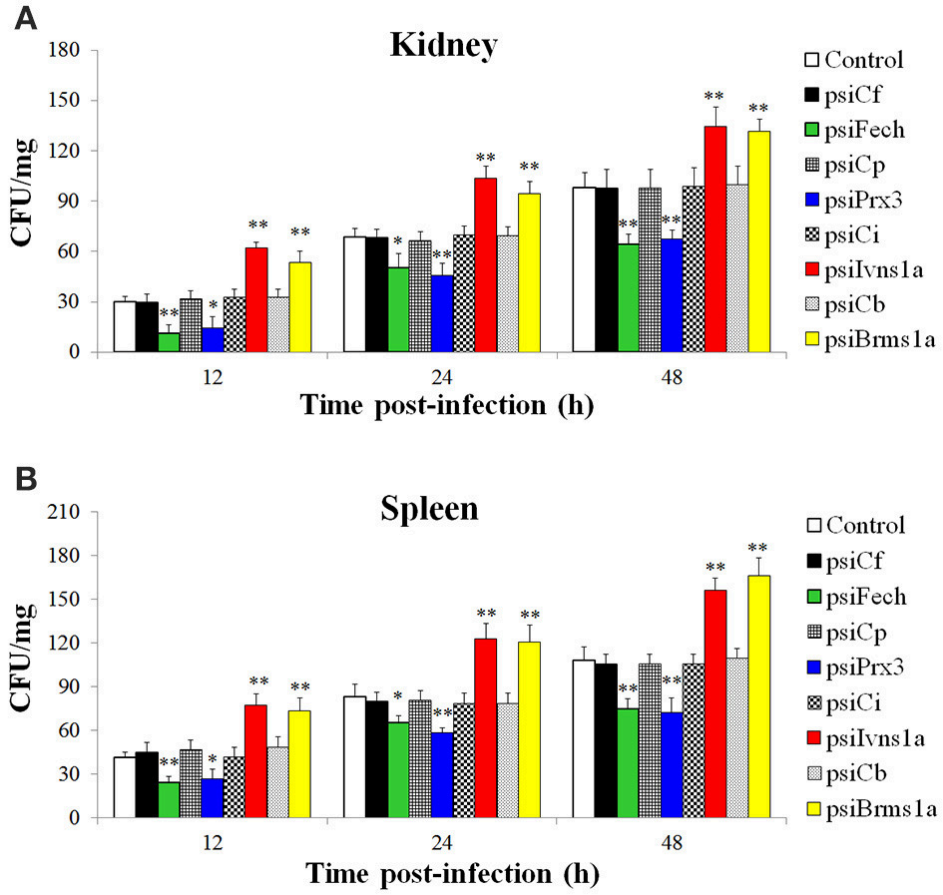
**Effect of ***Fech***, ***Prx3***, ***Brms1a***, and ***Ivns1a*** knockdown on ***Edwardsiella tarda*** infection**. Zebrafish were administered with psiFech, psiPrx3, psiBrms1a, psiIvns1a, psiCf, psiCp, psiCb, psiCi, or PBS (control). The fish were challenged with *E. tarda* at 3 days post-plasmid administration. Bacterial loads in kidney **(A)** and spleen **(B)** were determined at various time points after infection. Data are the means of three independent experiments and presented as means ± SEM. ^*^P < 0.05, ^**^P < 0.01.

The fish administered with psiFech, psiPrx3, psiBrms1a, psiIvns1a, and the control vectors were challenged with *E. tarda*, and the bacterial loads in kidney and spleen were determined by plate count at 12, 24, and 48 hpi. The results showed that in both tissues and at all examined time points, the bacterial numbers in fish administered with psiFech and psiPrx3 were significantly lower than those in the control fish, whereas the bacterial numbers in fish administered with psiBrms1a and psiIvns1a were significantly higher than those in the control fish (Figure 5). In contrast, the bacterial numbers in fish administered with psiCf, psiCp, psiCb, and psiCi were comparable to those in the control fish.

### Caspase 3 activity in fish with gene-overexpression and gene-knockdown

**Figure 6:**
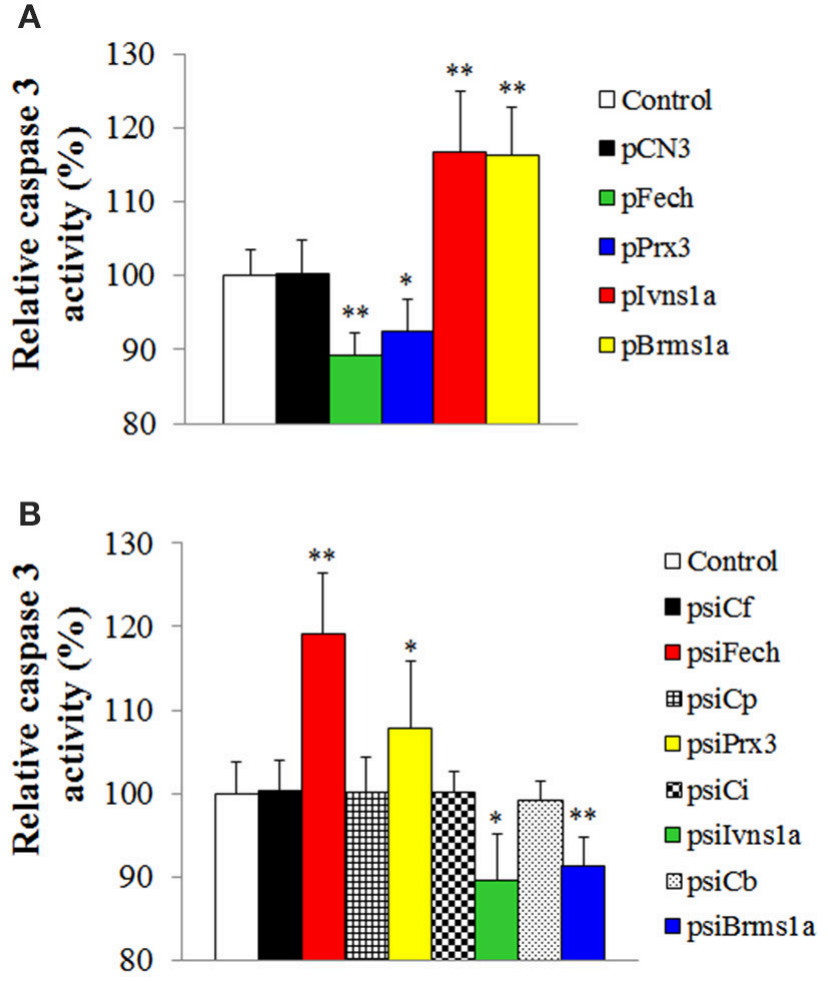
**Effect of ***Fech***, ***Prx3***, ***Brms1a***, and ***Ivns1a*** overexpression and knockdown on caspase 3 activity. (A)** Zebrafish were administered with pFech, pPrx3, pBrms1a, pIvns1a, pCN3, or PBS (control), and the activity of caspase 3 in spleen was determined at 3 days post-plasmid administration. **(B)** psiFech, psiPrx3, psiBrms1a, psiIvns1a, psiC1f, psiCp, psiCb, psiCi, or PBS (control), and the activity of caspase 3 in spleen was determined at 3 days post-plasmid administration. Data are the means of three independent experiments and presented as means ± SEM. ^*^P < 0.05, ^**^P < 0.01.

To examine whether *Fech, Prx3, Brms1a*, and *Ivns1a* overexpression and knockdown affected the apoptosis status of the fish, the activity of caspase 3 was determined in the fish. The results showed that in zebrafish administered with pFech and pPrx3, caspase 3 activity was significantly reduced compared to the control fish, whereas in zebrafish administered with psiBrms1a and psiIvns1a, caspase 3 activity was significantly increased compared to the control fish (Figure 6A). In zebrafish administered with psiFech and psiPrx3, caspase 3 activity was significantly higher than that in the control fish, whereas in zebrafish administered with psiBrms1a and psiIvns1a, caspase 3 activity was significantly lower than that in the control fish (Figure 6B). Fish administered with psiCf, psiCp, psiCb, and psiCi showed no significant alteration in caspase 3 activity compared to control fish.

## Discussion

In the past decade, zebrafish has become an important model organism for the study of infectious disease, especially host-pathogen interaction (Pressley et al., 2005; Lieschke and Currie, 2007; Meeker and Trede, 2008; Sullivan and Kim, 2008; Ellett and Lieschke, 2010; van Soest et al., 2011; Yang et al., 2012; Ablain and Zon, 2013). In this study, we utilized the zebrafish system to investigate the effect of *E. tarda* on apoptosis. It is known that *E. tarda* can survive in Japanese flounder FG-9307 cells, fathead minnow EPC cells, mice J774 macrophages, and human HEp-2 cells (Okuda et al., 2006, 2008; Wang et al., 2013). In our study, we found that following incubation with ZF4 cells, *E. tarda* was detected inside the cells with an amount that increased with time, suggesting that *E. tarda* invaded into ZF4 and replicated therein.

An increasing number of pathogens are now known to inhibit apoptosis, which is considered to play a critical role in the pathogenesis of the microbes (Faherty and Maurelli, 2008; Rudel et al., 2010; Raymond et al., 2013; Siamer and Dehio, 2015). For instance, *Shigella flexneri* inhibits apoptosis of HeLa cells and T84 cells by preventing caspase 3 activation (Faherty and Maurelli, 2008), and *Neisseria gonorrhoeae* inhibits apoptosis by preventing cytochrome *c* release through the secretion of a bacterial product (Morales et al., 2006; Faherty and Maurelli, 2008). A previous report showed that *E. tarda*-infected flounder FG-9307 cells failed to form apoptotic bodies and DNA ladder (Wang et al., 2013). In the case of our study, we found that *E. tarda*-infected ZF4 cells displayed negative biochemical hallmarks of apoptosis, including nuclear chromatin condensation, DNA fragmentation, apoptotic bodies, and activation of caspase 3/8/9. These results suggested that the apoptosis pathway in *E. tarda*-infected ZF4 cells was likely not activated.

Reports have indicated that in zebrafish, the expression of immune genes were altered in response to the infection of a live attenuated *E. tarda* strain (Yang et al., 2012), and that the mRNA levels of inflammatory cytokines (interleukin-1β and tumor necrosis factor-α) were significantly upregulated in *E. tarda*-infected fish (Pressley et al., 2005). In our study, transcriptome analysis revealed a global expression profile of *E. tarda*-regulated genes including those involved in apoptosis, which was confirmed by qRT-PCR analysis. Okuda et al. (2006) have reported that *E. tarda* infection of murine macrophages was accompanied by upregulation of the anti-apoptotic genes *Bcl2a1a, Bcl2a1b, cIAP-2*, and *TRAF1*, which protect macrophages from staurosporine-induced apoptosis. Consistently, we found that in *E. tarda*-infected ZF4 cells, the expressions of anti-apoptotic genes were significantly upregulated, whereas the expressions of pro-apoptotic genes were significantly downregulated. These results were in agreement with the absence of apoptotic markers in *E. tarda*-infected ZF4 and suggested that *E. tarda* likely had a negative effect on host cell apoptosis.

Most of the apoptotic genes identified in fish are putative. Of the anti-apoptotic genes upregulated by *E. tarda, Fech* (encoding ferrochelatase) and *Prx3* (encoding peroxiredoxin 3) had not been previously reported in fish. Ferrochelatase is a key enzyme that catalyzes the conversion of protoporphyrin IX (PpIX) to heme (Teng et al., 2011). Previous studies in mammalian cells showed that inhibition of ferrochelatase led to increased PpIX accumulation and enhanced apoptosis of PC-3 cells (Amo et al., 2009); Peroxiredoxin 3 is a critical mitochondrion-specific H_2_O_2_-scavenging enzyme (Godahewa et al., 2015), and its depletion resulted in increased intracellular levels of H_2_O_2_, which sensitized cells to the induction of apoptosis by staurosporine or TNF-α (Chang et al., 2004). In the present study, we found that following *E. tarda* inoculation, fish administered with pFech and pPrx3 exhibited significantly increased bacterial burdens and reduced caspase 3 activity in tissues, suggesting that *Fech* and *Prx3* overexpression inhibited apoptosis, which allowed the invading pathogen to replicate intracellularly. In line with these observations, fish with *Fech* and *Prx3* knockdown exhibited significantly reduced bacterial loads and increased caspase 3 activity, suggesting that interference with *Fech* and *Prx3* expression promoted apoptosis and enhanced the ability of zebrafish to clear the invading pathogen. These results also indicated for the first time that *Fech* and *Prx3* are associated with apoptosis in teleost.

Of the pro-apoptotic genes downregulated by *E. tarda, Brms1a* (encoding breast cancer metastasis suppressor 1 a) is a metastasis suppressor that was first identified in breast cancer (Hurst et al., 2009). In humans, expression of *Brms1* in SK-Hep1 cells sensitized the cells to apoptosis induced by serum deprivation or anoikis (Wu et al., 2012), while knockdown of endogenous *Brms1* in Hep3B cells suppressed apoptosis (Wu et al., 2012). *Ivns1a* (encoding influenza virus NS1A binding protein a) is another pro-apoptotic gene, whose expression has been shown to be sufficient to induce apoptosis in MDCK and HeLa cells (Schultz-Cherry et al., 2001; Zhirnov et al., 2002). In our study, fish with *Brms1a* and *Ivns1a* overexpression exhibited significantly reduced bacterial loads and increased caspase 3 activity, whereas the opposite was true in fish with *Brms1a* and *Ivns1a* knockdown. These results indicated that, as observed in mammalian systems, *Brms1a* and *Ivns1a* were required for apoptosis in zebrafish and consequently targeted by *E. tarda*.

In conclusion, we demonstrated for the first time that apoptosis is essential for teleost to combat *E. tarda* invasion, and, as a result, *E. tarda* inhibits apoptosis by regulating the genes involved in the apoptotic process. Hence, prevention of apoptosis is a virulence strategy of *E. tarda* that enables the pathogen to survive and replicate inside host cells. In addition, our study also provided the first evidence that *Fech, Prx3, Brms1a*, and *Ivns1a* are involved in apoptosis in teleost.

## Author contributions

ZZ and LS designed the study; ZZ performed experiments; ZZ and LS analyzed data and wrote the article.

### Conflict of interest statement

The authors declare that the research was conducted in the absence of any commercial or financial relationships that could be construed as a potential conflict of interest.

## Supplementary material

Additional file 1 **Annotation results of 958 differentially expressed genes**.

Table S1 **PCR primers used in this study**.

Table S2 **Expression of apoptosis-associated genes in ZF4 before and after ***Edwardsiella tarda*** infection**. ZF4 cells were treated with *E. tarda* for different hours, and the expression of 23 apoptosis-associated genes was determined by quantitative real time RT-PCR. The value of each time point after infection is shown as fold change in comparison 0 hpi.

Figure S1 **Volcano plot of differentially expressed genes**. For every gene, the ratio of expression levels in the *Edwardsiella tarda*-infected cells over that in the control cells was plotted against the -Lg (*p*-value). The vertical lines indicate the two fold change threshold, and the horizontal lines indicate *p*-value = 0.01.

Figure S2 **The top 10 KEGG pathways of differentially expressed genes**.

Figure S3 **Effect of ***Edwardsiella tarda*** on the caspase activity of ZF4 cells**. ZF4 cells were infected with *E. tarda* or treated with cisplatin, and the control cells were treated with PBS. At 12 and 24 h post infection (hpi), the activity of caspase 3, 8, and 9 was measured. For convenience of comparison, the activity of the control cells was defined as 1. Data are the means of three independent experiments and presented as means ± SEM. NS, no significance.

Figure S4 **Detection of plasmids (A) and expression of plasmid-derived immune genes (B) in zebrafish. (A)** Zebrafish were administered with pCN3 (lanes 7 to 11), pFech (lane 12), pPrx3 (lane 13), pBrms1a (lane 14), pIvns1a (lane 15), and PBS (lanes 2 to 6). At 3 d post-plasmid administration, DNA was extracted from spleen and used for PCR with primers specific to pFech (lanes 3, 8, and 12), pPrx3 (lanes 4, 9, and 13), pBrms1a (lanes 5, 10, and 14), pIvns1a (lanes 6, 11, and 15, and pCN3 (lanes 2 and 7). **(B)** Zebrafish were administered with pCN3 (lane 6 to 9), pFech (lane 10), pPrx3 (lane 11), pBrms1a (lane 12), pIvns1a (lane 13), and PBS (lanes 2 to 5). At 3 d after plasmid administration, RNA was extracted from spleen and used for RT-PCR with primers specific to plasmid-derived Fech (lanes 2, 6, and 10), Prx3 (lanes 3, 7, and 11), Brms1a (lanes 4, 8, and 12), and Ivns1a (lanes 5, 9, and 13), or, as an internal control, with primers specific to β-actin (lower panel). Lane 1 of all panels, DNA molecular weight markers.

Figure S5 **Knockdown of ***Fech, Prx3, Brms1a***, and ***Ivns1a*** expression by RNAi**. Zebrafish were administered with psiFech, psiPrx3, psiBrms1a, psiIvns1a, psiCf, psiCp, psiCb, psiCi, or PBS (control). At 3 d **(A)** and 5 d **(B)** post-plasmid administration, the expression levels of *Fech, Prx3, Brms1a*, and *Ivns1a* in kidney (**Aa** and **Ba**) and spleen (**Ab** and **Bb**) were determined by quantitative real time RT-PCR. In each case, the expression level of the control fish was set as 1. Data are the means of three independent experiments and presented as means ± SEM. ^*^*P* < 0.05, ^**^*P* < 0.01.
