# Supplementary material for: Edwardsiella tarda-Induced Inhibition of Apoptosis: A Strategy for Intracellular Survival
Source: Front Cell Infect Microbiol. 2016 Jul 14;6:76. doi: 10.3389/fcimb.2016.00076 (PMC4943942; doi:10.3389/fcimb.2016.00076)
Supplement: Table S1 — PCR primers used in this study. [file Table1.DOC]

**Supplementary material**

**Table S1.**

| Primers | Sequence (5’-3’) ^a^ | Gene | Accession no. |
| --- | --- | --- | --- |
| Actin-RT-F | CCATCGGCAATGAGCGTTTC | β-actin | AAC13314.1 |
| Actin-RT-R | TGGCATACAGGTCCTTACGG |
| AIF-RT-F | TGGTGCCAGGGTGTTGATTAT | AIF | NP_956396.2 |
| AIF-RT-R | TACTCCTTTCTTTGCCGTTCC |
| Bad-RT-F | TGATAAAGGGCAGATGAAGAG | Bad | NP_001257524.1 |
| Bad-RT-R | GACTCCGCATCAGACTCTTTG |
| Bax-RT-F | CTCTAATCTTCAGCCGACTCA | Bax | XP_001343654 |
| Bax-RT-R | GACAAGGCGACAGGCAAAGT |
| Bcl-2-RT-F | CATTCTTCGAGTTTGGTGGGA | Bcl-2 | XP_001341214 |
| Bcl-2-RT-R | TGGCCCGTTCAGGTAGTCAGT |
| Bcl-XL-RT-F | TAGACGCAGTGAAGGAGGC | Bcl-XL | NP_571882 |
| Bcl-XL-RT-R | GTGGCGGGTGTGATGTGGA |
| Bid-RT-F | TATAGAAACCGACGGACAAAG | Bid | NP_001073295 |
| Bid-RT-R | ACTCTGCCGCCATTTCTCTC |
| Brms1a-RT-F | AGTTCAGAAATGGACGACGAA | Brms1a | NM_001030102.2 |
| Brms1a-RT-R | CACTTCCTGTAGTTTGGCATC |
| Casp3-RT-F | TGTTGCTCAGTCACGGCGATG | Caspase 3 | NP_571952.1 |
| Casp3-RT-R | AGTGATGGGCAGCGGTCTCCT |
| Casp8-RT-F | ATGAAGGGGGAACGGAGAGGA | Caspase 8 | XP_685430.4 |
| Casp8-RT-R | AAAGCCCAGCCACTCAAACAC |
| Casp9-RT-F | ACAGAGCAAGGCAACCAGAC | Caspase 9 | NP_001007405 |
| Casp9-RT-R | CTCACGCAGGGAATCAAGAA |
| CytC-RT-F | TTCGGTCGCAAGACTGGTCAG | Cyt C | NP_001002068 |
| CytC-RT-R | GGAATGTATTTCTTTGGGTTCT |
| DFF40-RT-F | CCAGGGTGCTTTTGATGAGA | DFF40 | NP_919385.1 |
| DFF40-RT-R | GGCGAGAGCAGGAATGACAG |
| DFF45-RT-F | GGCGATGATGGAGGTGTTGGT | DFF45 | NP_001002631.1 |
| DFF45-RT-R | TGCTTGTGGATCGGACTGGTT |
| EndG-RT-F | TGCGAGTTCAAAGAGGACGAG | EndG | NP_001019385 |
| EndG-RT-R | ACTTGTGATTGGCTGCTGCTG |
| FADD-RT-F | GGTAAAAAGTGGCCCTCCGTT | FADD | XP_001923893 |
| FADD-RT-R | GCTCTCTGACCTGCTCCTCC |
| Fech-RT-F | GCAGCAGTCTGAACGCCATCT | Fech | XM_005170244.2 |
| Fech-RT-R | CACTCTATCAGCAGCGGGTGT |
| FLIP-RT-F | AGTGACCGCATCTCTTAGCAG | FLIP | NP_919380 |
| FLIP-RT-R | GGCCAGCAAATCTCCTCTCAG |
| IAP2-RT-F | AGGGTGGCGTGCTTTAGTTGT | IAP2 | NP_919376 |
| IAP2-RT-R | CGCCACCAGATAAAGGGATGT |
| Ivns1a-RT-F | CTCACCTTCGGCTACACCTT | Ivns1a | NM_199279.2 |
| Ivns1a-RT-R | TCCACCTGCGGCGATAAGTT |
| Prx3-RT-F | GGGCACTGCTGTTATCAATGG | Prx3 | NM_001013460.3 |
| Prx3-RT-R | CGTGAAACTCGTTGGCTTTGT |
| Rip1-RT-F | CGACGGTTTGACGGAGAAAGT | Rip3 | NP_001036815 |
| Rip1-RT-R | AGGAGGGCGTTGGGTTTGATG |
| TNF-R1-RT-F | GAGTGATGGTGGCAGTGGAG | TNF-R1 | NP_998355 |
| TNF-R1-RT-R | CGAAACGCTTGTGTTCTGTGA |
| Traf2-RT-F | AGACGTTGGGCTGGGATTGTA | Traf2 | XP_005165556 |
| Traf2-RT-R | AGCGTTCCACCTCTTTATTCA |
| Brms1a-OE-F | GATATCGCCACCATGCCAGTGCACTCGAGAGAA | Brms1a | NM_001030102.2 |
| Brms1a-OE-R | GATATCGGCATGTTTTACGGTGTATTTGC |
| Fech-OE-F | GATATCGCCACCATGGCGGTTTTAGGATGCGCGT | Fech | XM_005170244.2 |
| Fech-OE-R | GATATCGAGTTTCTGGCTGGAGAAGAAGGCT |
| Ivns1a-OE-F | GATATCGCCACCATGATTCCCAACGGATATTTGAT | Ivns1a | NM_199279.2 |
| Ivns1a-OE-R | GATATCTTGAGAAAAAACTCCAAACGCTA |
| Prx3-OE-F | GATATCGCCACCATGGCAGCCACCATCGGGAGAC | Prx3 | NM_001013460.3 |
| Prx3-OE-R | GATATCGTTGACCTTTTCGAAGTATTCCTTA |
| CNF1 | CTTGCGTTTCTGATAGGCACCTA |  |  |
| CNR1 | TGCGGGCCTCTTCGCTATT |  |  |
| His-R | GTGGTGGTGGTGGTGGTG |  |  |

^a^Underlined nucleotides are restriction site of the EcoRV enzyme.
