# Supplementary material for: Edwardsiella tarda-Induced Inhibition of Apoptosis: A Strategy for Intracellular Survival
Source: Front Cell Infect Microbiol. 2016 Jul 14;6:76. doi: 10.3389/fcimb.2016.00076 (PMC4943942; doi:10.3389/fcimb.2016.00076)
Supplement: Table S2 — Expression of apoptosis-associated genes in ZF4 before and after Edwardsiella tarda infection. ZF4 cells were treated with E. tarda for different hours, and the expression of 23 apoptosis-associated genes was determined by quantitative real time RT-PCR. The value of each time point after infection is shown as fold change in comparison 0 hpi. [file Table2.DOC]

**Supplementary material**

**Table S2.**

| **Gene** | **Relative gene expression**  **Log2 (fold change)** | | | | |
| --- | --- | --- | --- | --- | --- |
|  | 0 hpi | 6 hpi | 12 hpi | 24 hpi | 48 hpi |
| *Ferrochelatase, Fech* | 0.00 | 1.52** | 1.55** | 2.70** | 3.42** |
| *Peroxiredoxin 3, Prx3* | 0.00 | 0.98* | 1.47** | 2.64** | 3.27** |
| *Baculoviral IAP repeat containing 2, IAP2* | 0.00 | 0.08 | 1.08** | 1.94** | 2.20** |
| *CASP8 and FADD-like apoptosis regulator a, FLIP* | 0.00 | 0.88** | 1.08* | 1.25** | 1.84** |
| *B-cell CLL/lymphoma 2a, Bcl-2* | 0.00 | 0.83** | 0.86** | 0.96* | 1.58** |
| *Tnf receptor-associated factor 2b, TRAF2* | 0.00 | -1.38** | -1.07* | -0.64* | -0.28 |
| *BCL2-associated agonist of cell death b, Bad* | 0.00 | -1.06** | -0.80* | -0.69* | -0.60* |
| *Bcl2-like 1, Bcl-XL* | 0.00 | -0.67** | -0.78* | -0.68** | -0.77* |
| *Bcl2-associated X protein a, Bax* | 0.00 | -0.51* | -0.89* | -0.95** | -1.21* |
| *DNA fragmentation factor, 40 kD, beta subunit, DFF40* | 0.00 | -2.28* | -1.48* | -1.28** | -1.03* |
| *DNA fragmentation factor, 45 kD, alpha subunit, DFF45* | 0.00 | -1.14** | -1.11** | -1.06* | -0.87* |
| *Receptor (TNFRSF)-interacting serine-threonine kinase 1, Rip1* | 0.00 | -0.62** | -0.68* | -0.81* | -0.95** |
| *Apoptosis-inducing factor, mitochondrion-associated 1, AIF* | 0.00 | -0.86 | -0.86** | -0.90* | -1.13* |
| *Endonuclease G, EndG* | 0.00 | -1.02* | -1.11* | -1.24** | -1.22** |
| *Tumor necrosis factor receptor superfamily, member 1a, TNF-R1* | 0.00 | -1.55** | -1.33** | -1.00** | -1.10** |
| *Cytochrome c, somatic b, CytC* | 0.00 | -0.68* | -0.76** | -1.05** | -1.11** |
| *Caspase 8* | 0.00 | -1.62** | -2.17** | -2.36** | -2.37** |
| *Caspase 3* | 0.00 | -2.34* | -2.47** | -2.83** | -3.15** |
| *Caspase 9* | 0.00 | -1.66** | -2.74** | -2.75** | -2.81** |
| *Fas (tnfrsf6)-associated via death domain, FADD* | 0.00 | -1.09** | -1.55** | -1.60** | -3.18** |
| *BH3 interacting domain death agonist, Bid* | 0.00 | -1.79* | -2.46** | -2.74** | -3.34** |
| *Breast cancer metastasis-suppressor 1, Brms1a* | 0.00 | -0.54** | -1.78** | -2.64** | -3.36** |
| *Influenza virus NS1A binding protein a, Ivns1a* | 0.00 | -0.40* | -1.37** | -2.47** | -4.20** |
