# Supplementary figures and images for: Edwardsiella tarda-Induced Inhibition of Apoptosis: A Strategy for Intracellular Survival
Source: Front Cell Infect Microbiol. 2016 Jul 14;6:76. doi: 10.3389/fcimb.2016.00076 (PMC4943942; doi:10.3389/fcimb.2016.00076)

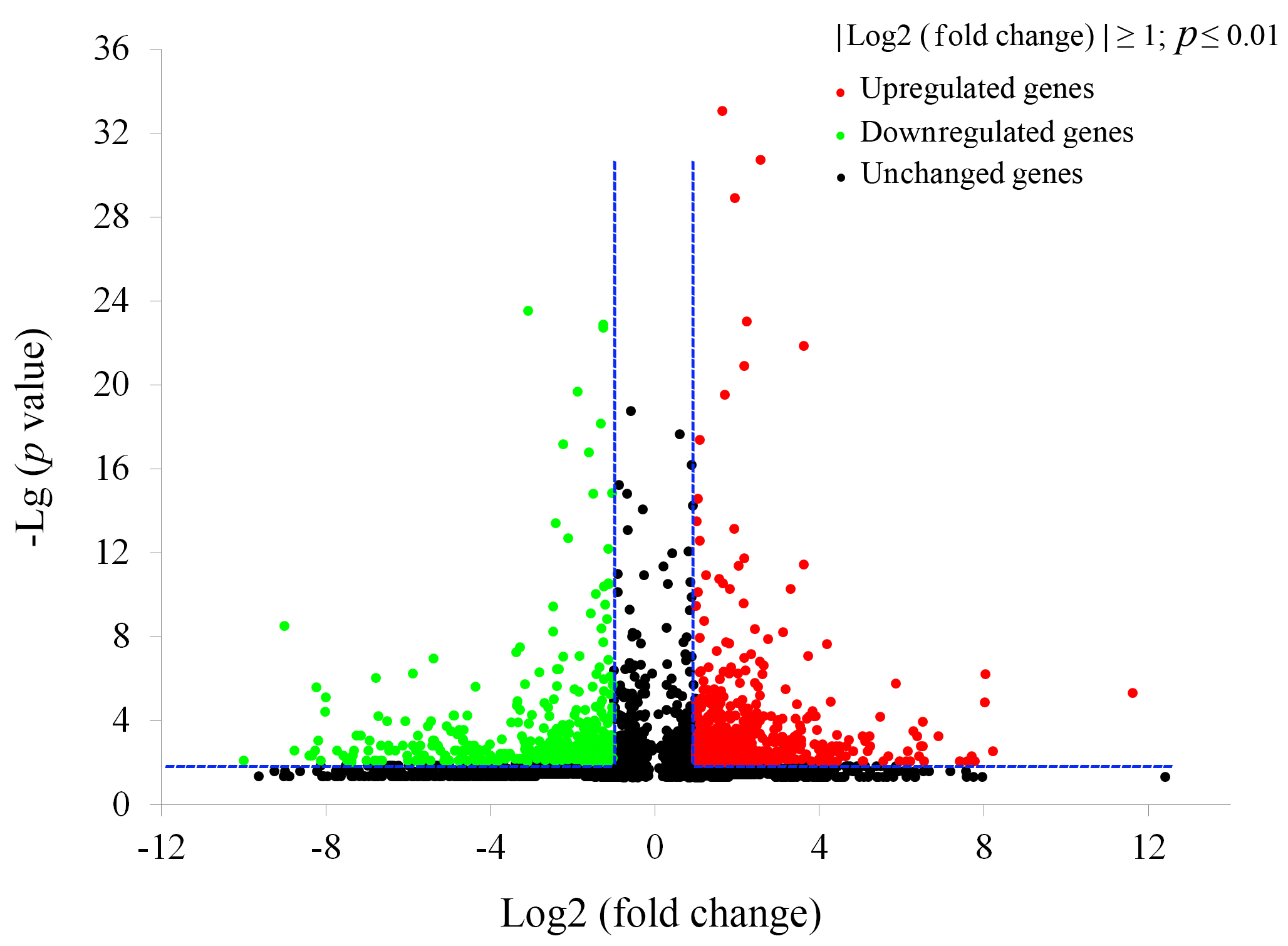

Supplement: Figure S1 — Volcano plot of differentially expressed genes. For every gene, the ratio of expression levels in the Edwardsiella tarda-infected cells over that in the control cells was plotted against the -Lg (p-value). The vertical lines indicate the two fold change threshold, and the horizontal lines indicate p-value = 0.01. [file Image1.TIF]

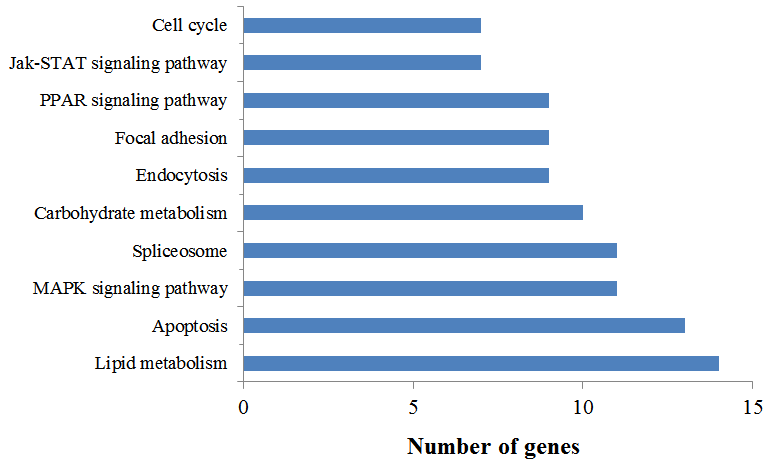

Supplement: Figure S2 — The top 10 KEGG pathways of differentially expressed genes. [file Image2.TIF]

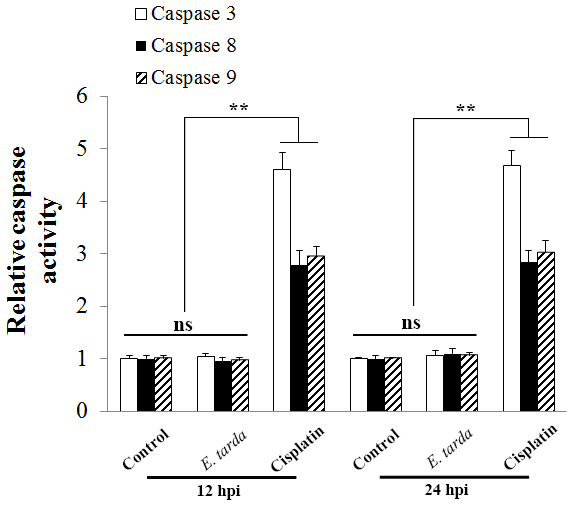

Supplement: Figure S3 — Effect of Edwardsiella tarda on the caspase activity of ZF4 cells. ZF4 cells were infected with E. tarda or treated with cisplatin, and the control cells were treated with PBS. At 12 and 24 h post infection (hpi), the activity of caspase 3, 8, and 9 was measured. For convenience of comparison, the activity of the control cells was defined as 1. Data are the means of three independent experiments and presented as means ± SEM. NS, no significance. [file Image3.TIF]

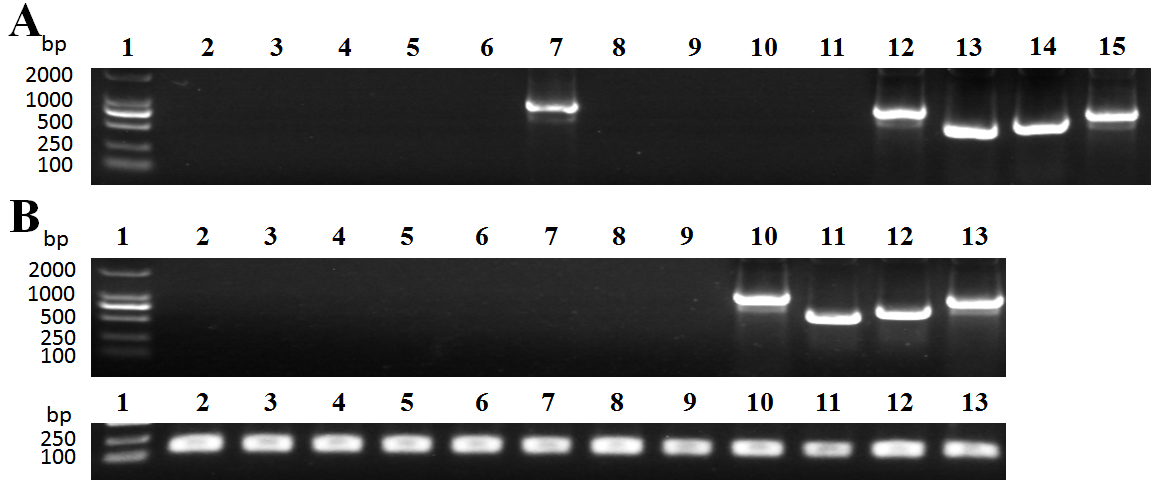

Supplement: Figure S4 — Detection of plasmids (A) and expression of plasmid-derived immune genes (B) in zebrafish. (A) Zebrafish were administered with pCN3 (lanes 7 to 11), pFech (lane 12), pPrx3 (lane 13), pBrms1a (lane 14), pIvns1a (lane 15), and PBS (lanes 2 to 6). At 3 d post-plasmid administration, DNA was extracted from spleen and used for PCR with primers specific to pFech (lanes 3, 8, and 12), pPrx3 (lanes 4, 9, and 13), pBrms1a (lanes 5, 10, and 14), pIvns1a (lanes 6, 11, and 15, and pCN3 (lanes 2 and 7). (B) Zebrafish were administered with pCN3 (lane 6 to 9), pFech (lane 10), pPrx3 (lane 11), pBrms1a (lane 12), pIvns1a (lane 13), and PBS (lanes 2 to 5). At 3 d after plasmid administration, RNA was extracted from spleen and used for RT-PCR with primers specific to plasmid-derived Fech (lanes 2, 6, and 10), Prx3 (lanes 3, 7, and 11), Brms1a (lanes 4, 8, and 12), and Ivns1a (lanes 5, 9, and 13), or, as an internal control, with primers specific to β-actin (lower panel). Lane 1 of all panels, DNA molecular weight markers. [file Image4.TIF]

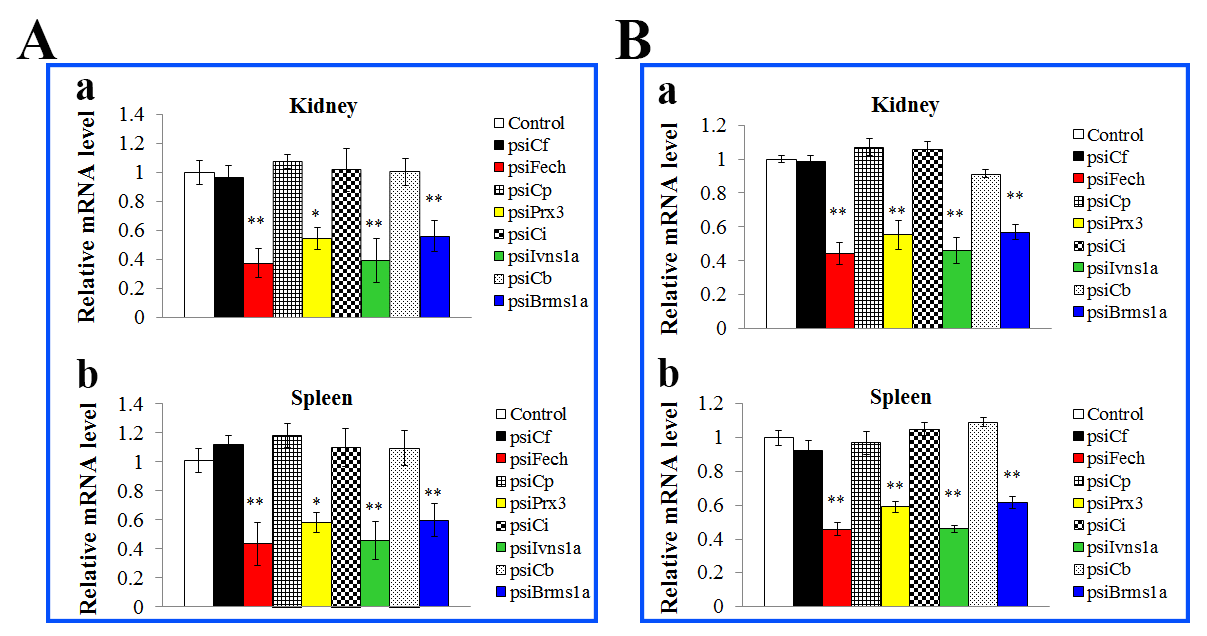

Supplement: Figure S5 — Knockdown of Fech, Prx3, Brms1a, and Ivns1a expression by RNAi. Zebrafish were administered with psiFech, psiPrx3, psiBrms1a, psiIvns1a, psiCf, psiCp, psiCb, psiCi, or PBS (control). At 3 d (A) and 5 d (B) post-plasmid administration, the expression levels of Fech, Prx3, Brms1a, and Ivns1a in kidney (Aa and Ba) and spleen (Ab and Bb) were determined by quantitative real time RT-PCR. In each case, the expression level of the control fish was set as 1. Data are the means of three independent experiments and presented as means ± SEM. *P < 0.05, **P < 0.01. [file Image5.TIF]
